# Editor's choice: Infectious disease are still dominating the African scene

**DOI:** 10.4314/ahs.v24i3.1

**Published:** 2024-09

**Authors:** James K Tumwine

Welcome to this September 2024 issue of *African Health Sciences*, in which we bring you very interesting papers on; infections, communicable disease, sexual reproductive health, and systems. The papers on infectious diseases include: a systematic review on HIV virologic failure^1^; impact of lockdowns on ART adherence in Zimbabwe^2^; community engagement in Uganda^3^, antibiotic sensitivity of uro-pathogens^4^; extended spectrum and beta lactamase^5^. The papers also touch on hepatitis C^6^; antibiotic resistance in Iraq^7^; flow cytometry for detection of antibiotic resistance^8^; severe pneumonia^9^, and molecular biology of TB^10^. The next infectious disease papers are on neonatal tetanus in South Sudan^11^, acute kidney injury in malaria^12^, malaria in Kenya^13^,^14^, and aflatoxin in maize in Tanzania 15. The next set of papers is on cancer^16^-^27^; sexuality and reproductive health^28^-^32^. We also have papers on child health^33^-^37^; non-communicable diseases^38^-^48^, and others^49^-^52^.

The set of cancer papers include Dutta's paper on antiinflammatory activity on leukemic cells^16^, JAK2 and neoplasms in Morocco^17^; late diagnosis of prostate cancer in Uganda^18^, and breast cancer in Zimbabwe^19^. The other cancer papers touch on Semaphorin 6D and its role in spread of gastric cancer^20^; abdominal visceral fat and risk of endometrial cancer^21^; FNA and ultrasonography in thyroid cancer^22^; MiRNA and progression of non-small cell lung cancer^23^; early diagnosis of gastric cancer^24^; chronic myeloid leukemia^25^; and finally, childhood cancer survivors^26^.

The treatise on sexual reproductive health and Pediatric conditions includes work on abortion^27^, postpartum anxiety^28^; women infertility and vitamin D^29^; guidelines for management of women infertility^30^; surgery for gynecological oncology^31^; nutrition among non-pregnant women in Zimbabwe^32^; diarrhea outbreaks in Ethiopia^33^; and calprotectin and breastfeeding^34^; complementary feeds in Uganda^35^; congenial malformations^36^; cleft lip and palate^37^.

Now over to non-communicable diseases. We have papers on diabetes mellitus^38^,^39^; hypertension 40; stroke^41^; treatment of migraine^42^; orthopedic surgery^43^; and alopecia^44^; red cell alloimmunization and sickle cell disease^45^; substance abuse in Tanzania^46^; dopamine receptors in mice^47^; plaque burden^48^; traditional medicine^49^; effect of mobile phones in drug education^50^; dissemination of research findings^51^; and management of nocturnal hemoglobinuria^52^.

Please enjoy this September 2024 issue of African Health Sciences. Happy reading!
